# Simulated patients’ role-portrayal in the clinical skills part of the Swiss federal licensing exam is of high quality and improves further over time as measured with the FAIR OSCE instrument 

**DOI:** 10.3205/zma001736

**Published:** 2025-02-17

**Authors:** Kai P. Schnabel, Daniel Bauer, Felix M. Schmitz, Tanja Hitzblech, Beate G. Brem

**Affiliations:** 1 University of Bern, Institute for Medical Education, Bern, Switzerland

**Keywords:** OSCE, objective structured clinical examination, standardization, standardized patien, simulated patient, high-stakes examinations, licensing exam, patient simulation

## Abstract

**Introduction:**

Simulation-based teaching and assessment are integral to education in the health professions, with simulated patients (SP) being a widely accepted strategy. Ensuring high-quality SP role-portrayal is crucial for the authenticity and standardization of assessments, particularly in high-stakes exams like the Swiss Federal Licensing Examination (FLE).

The study assesses the quality of SP role-portrayal over consecutive instances of the Swiss FLE. We hypothesized that the quality of role-portrayal improves over time.

**Methods:**

The study employed the FAIR OSCE instrument to assess SP role-portrayal in five consecutive FLE exams from 2016 to 2021. The instrument, developed between 2011 and 2014, includes four categories: introduction, delivery of information, portrayal, and others. Data analysis involved retrospective examination of FAIR OSCE ratings, calculating item scores, and overall mean scores for each exam year.

**Results:**

The study involved 37 SP educators observing 1803 SP-candidate interactions across five exam sites. Results demonstrated a continuous improvement in SP role-portrayal over the five-year period, with significant differences between 2016 and subsequent years. The overall mean scores of SP role-play ratings increased steadily, indicating a positive trend in SP performance.

**Discussion:**

The findings supported the hypothesis of continuous improvement of SP role-portrayal within the Swiss FLE. The quality of role portrayal not only improved consistently but also maintained a very high level, with no items on the FAIR OSCE instrument rated “do not agree” more than 5% of the time. This suggests that SPs role play aligned well with case scripts, reflecting the intended authenticity and standardization of assessments.

Limitations were acknowledged, including potential bias in local SP educators rating their own SPs and the study’s sole focus on SP role-portrayal. While the findings contribute to understanding SP effectiveness in standardized, high-stakes clinical exams, the study did not scrutinize other potential sources of variance.

**Conclusion:**

In conclusion, the research demonstrated a continuous improvement and high quality of SP role-portrayal in the Swiss FLE over five years. Well-trained SPs, assessed using the FAIR OSCE instrument, play a crucial role in maintaining the standardized and high-quality nature of clinical skills exams in a high-stakes context. Further research could explore additional factors influencing overall exam quality and address potential biases in SP educator ratings.

## Zusammenfassung

**Einleitung:**

Simulationsbasierte Lehre und Prüfungen sind integraler Bestandteil der Ausbildung in den Gesundheitsberufen, wobei der Einsatz von Simulationspersonen (SP) eine weithin akzeptierte Strategie darstellt. Die Sicherstellung einer qualitativ hochwertigen SP-Rollendarstellung ist entscheidend für die Authentizität und Standardisierung von Prüfungen, insbesondere bei karriere-relevanten summativen Prüfungen wie der eidgenössischen Prüfung in Humanmedizin Clinical Skills (EPCS) der Schweiz.

Die Studie beurteilt die Qualität der SP-Rollendarstellung über mehrere aufeinanderfolgende EPCS. Wir stellten die Hypothese auf, dass sich die Qualität der Rollendarstellung mit der Zeit verbessert.

**Methoden:**

In der Studie wurde das FAIR-OSCE-Instrument verwendet, um die SP-Rollendarstellung in fünf aufeinanderfolgenden EPCS von 2016 bis 2021 zu bewerten. Das Instrument, das zwischen 2011 und 2014 entwickelt wurde, umfasst vier Kategorien: Einführung, Informationsvermittlung, Rollendarstellung und weitere Elemente. Die Datenanalyse umfasste die retrospektive Überprüfung der FAIR-OSCE-Bewertungen, die Berechnung der Item-Scores und der durchschnittlichen Gesamtpunktzahl für jedes Prüfungsjahr.

**Ergebnisse:**

An der Studie nahmen 37 SP-Expertinnen und Experten teil, die 1803 SP- Interaktionen mit Prüflingen an fünf Prüfungsstandorten beobachteten. Die Ergebnisse zeigten eine kontinuierliche Verbesserung der SP-Rollendarstellung über den Fünfjahreszeitraum, mit signifikanten Unterschieden zwischen 2016 und den Folgejahren. Die durchschnittlichen Gesamtwerte der SP-Rollenspielbewertungen stiegen stetig an, was auf einen positiven Trend in der SP-Leistung hindeutet.

**Diskussion:**

Die Ergebnisse stützten die Hypothese einer kontinuierlichen Verbesserung der SP-Rollendarstellung innerhalb der EPCS. Die Qualität der Rollendarstellung verbesserte sich nicht nur durchweg, sondern hielt sich auch auf einem sehr hohen Niveau, wobei kein Item auf dem FAIR-OSCE-Instrument in mehr als 5% der Fälle mit „stimme nicht zu“ bewertet wurde. Dies deutet darauf hin, dass die SP-Rollendarstellungen gut mit den Fallskripten übereinstimmten, was die beabsichtigte Authentizität und Standardisierung der Bewertungen widerspiegelt.

Ein möglicher Bias bei der Bewertung der eigenen SP durch lokale SP-Experten und der ausschliessliche Fokus der Studie auf die Rollendarstellung von SP müssen hier einschränkend genannt werden. Während die Ergebnisse zum Verständnis der Effektivität von SP bei standardisierten, bestehensrelevanten klinisch-praktischen Prüfungen beitragen, wurden in der Studie andere potenzielle Varianzquellen nicht untersucht.

**Schlussfolgerung:**

Zusammenfassend lässt sich sagen, dass die Studie über fünf Jahre hinweg eine kontinuierliche Verbesserung und die hohe Qualität der SP-Rollendarstellung in der EPCS zeigte. Gut ausgebildete SPs spielen, gemessen mit dem FAIR-OSCE-Instrument, eine entscheidende Rolle für die gleichbleibend hohe Qualität und Standardisierung bei karriereentscheidenden klinischen Kompetenzprüfungen. Weitere Forschung könnte zusätzliche Faktoren untersuchen, die die Gesamtqualität der Prüfungen beeinflussen, und potenzielle Bias bei den Bewertungen von SP-Expertinnen und -Experten untersuchen.

## Introduction

Simulation-based teaching and assessment are crucial in health professions education and assessment [1], [2]. Among different simulation-related options such as the use of mannequins, full-scale simulators and computer-based applications, employing simulated patients (aka simulated participants, SP) is a proven strategy for delivering simulation scenarios [2], [3]. First introduced in the 1960s [4], [5], working with SPs in learning and assessment contexts is nowadays widely accepted and applied by many medical schools all over the world [6]. Objective Structured Clinical Examinations (OSCEs) [7] regularly employ SPs, in which medical students interact with SPs portraying pre-defined patient cases, while examiners rate the students’ performance. The quality of the SPs’ role-portrayal is of critical importance to ensure fair and valid exams, above all in terms of authenticity and standardization [8]: while an *insufficiently authentic* role-portrayal limits the representativeness of the task put before the student, *insufficiently standardized* role-portrayal limits the comparability of their tasks and thus exam fairness, both are threats to the exam’s validity [2], [9], [10]. It is the role of SP educators to ensure their role-portrayal has a high level and good balance of authenticity and standardization [11], [12]. 

Switzerland introduced the new Federal Licensing Examination in (FLE) in 2011 [13], with a written exam and an OSCE that graduates of the medical Masters programs take in order to achieve licensing as physicians. An increasing number of candidates (medical students and candidates from non-European countries, seeking licensure in Switzerland) have to be assessed in the FLE with over a thousand taking the test in 2021 [14]. Several measures were put in place to ensure the highest quality in the development and delivery of this assessment [15], [16], [17]. The quality of the overall FLE is mirrored, e.g., with Cronbach’s alpha of above 0.84 as indicator of measurement reliability) [17].

To ensure each and every scenario’s SPs with their diverse individual backgrounds and acting experience are amalgamated into homogenous groups that portray their roles well over the whole exam day, a protocol for training and quality assurance was put in place. This includes 5 days of standardization meetings on a federal level to establish a common understanding of the scripts and scenarios and how to put them into practice and generating videographic material depicting the prototypical realization of every scenario (e.g., standardization of pain reactions, application of moulage [18], [19]). SP educators and SPs from their local program then use that material in two spaced trainings session of 2 hrs each to ensure authentic and standardized role portrayal during the exam [17]. During the exam, quality of SPs’ role portrayal (and other aspects of the exam) is controlled via live observation, e.g., via one-way mirrors or via audio-visual feed into a control room. 

Relying on an assessment format as complex and resource-intensive as the OSCE to make decisions that can impact both patients’ safety (candidates that pass as false-positive) but also test-takers’ careers (candidates that fail as false-negative) warrants an analysis to see if SP role-portrayal in high-stakes OSCE can be performed at high levels and continuously. Thus, the aim of the present study was to assess the quality of simulated patients’ (SP) role-portrayal in Swiss high-stakes clinical exams over time. We hypothesized that the quality of SP role-portrayal in the Swiss FLE continues to improve over time. 

## Methods

In order to determine if SP role-portrayal in high-stakes exams is performed at high levels in general and over time, we conducted a cohort study with five Swiss SP programs involved in the delivery of the OSCE within the FLE.

### Setting

The OSCEs were conducted at the time of this analysis at 5 sites (Basel, Bern, Geneva, Lausanne and Zurich), simultaneously in French and German, over three days, each run covering 12 stations, and almost all of which employed SPs (comprehensive data about the exam is available in attachment 1 ). 

### Procedure

To determine if OSCE-related SP role-portrayal is performed at high levels and consistently so, SP role-portrayal was assessed during the OSCEs within the FLE from 2016 to 2021, representing five consecutive exams as the 2020 clinical skills exam was not administered due to the COVID-19 pandemic. These five exams assessed over 5000 candidates’ clinical competencies, for which SPs were employed to deliver 70 parallel runs in total at 12 stations each. Data on these SPs’ role-portrayal were collected at all five sites over the five exams in random observations, via direct observation or live-stream as measured with the FAIR OSCE instrument. Raters were the local SP trainer (1-5 at each site) and one federal observer. The SP trainers were either physicians or experienced health professionals or artists with 2-20 years’ experience in the field and members of the federal SP trainer team. 

### Instrument

The FAIR OSCE instrument was developed between 2011 and 2014 by an interfaculty board of Swiss SP educators attempting to assess SP role-portrayal using *case-independent* observable criteria. It is based on the available literature and underwent a consensus-building process among the interfaculty board, supporting its content validity [15]. The instrument was additionally used in SP trainings as well as delivery of local formative and summative OSCEs at the faculty of Bern and has proven its feasibility and usability [15], [20]. 

The instrument encompasses four categories:

1. introduction (3 items),
2. delivery of information (7 items),
3. portrayal (7 items), and
4. others (3 items).

The items are assessed on a scale with the points *-1=do not agree, +1=agree, +2=completely agree*, and *not applicable*. (cf. attachment 2 ) 

### Data analysis

FAIR OSCE ratings from 2016 to 2021 were analysed retrospectively. Item scores were calculated to highlight the quality of SP role-portrayal within each year’s exam, and overall mean scores were calculated for each of the 5 exams (2016 to 2021, items rated as “not applicable” were excluded), i.e. all ratings derive from all sites administering the examination in a given year.

Statistical differences between examination years were calculated with one-way ANOVA. Effect sizes were calculated using eta squared with *η^2^~*0.01 for small, *η^2^~*0.06 for medium and *η^2^*>=0.14 for large effects. Post hoc tests were calculated using Bonferroni tests for each possible group comparison.

### Ethics approval

Ethics approval for this study was not necessary in accordance with Swiss legislation (chapter 1, article 2 of the 2011 Swiss Human Research Act). Ethics principles according to the WMA Helsinki declaration and good scientific practices were followed throughout.

## Results

During the 5 OSCEs between 2016 and 2021, totalling *N*=37 different SP educators (approximately 2-4 per site and year, mostly the same SP educators over the years) observed a total of *N*=1803 SP-candidate interactions (Basel *n*=247, Bern *n*=419, Geneva *n*=357, Lausanne *n*=467, and Zurich *n*=313) and rated the SP role-portrayal.

The number of observations increased from an initial 168 observations in 2016 to a maximum of 499 observations in 2021. 

To exemplify, table 1 (Tab. 1) shows the rating results for the OSCE 2021, based on 499 independent observations.

The overall mean scores of the SP role-play ratings increased continuously over the years (see table 2 (Tab. 2)). The ANOVA showed that at least one increasement across the years was of statistical significance (*F*=9.2, *p*<0.001; *η^2^*=0.02). The appropriate Bonferroni post hoc tests revealed that the results from 2018-2021 were significantly better compared to 2016 (cf. table 2 (Tab. 2)). 

## Discussion

In terms of our hypothesis, we were able to demonstrate that the quality of the SP role-portrayal increased continuously over the 5 years analyzed, significantly so when comparing data from 2021 versus 2016 and 2017, 2018 and 2017 versus 2016. 

This means we were able to confirm the initial hypothesis of increasing quality.

The quality of the role portrayal not only improved continuously but also was at a very high level with no items on the FAIR OSCE instrument rated *“do not agree”* more often than in 5% of random observations in all four categories, implying role-portrayal was as intended by the case scripts. Both these results can be seen as indicators both the exam and SPs employed are of the highest quality. It is also an indicator that the FAIR OSCE instrument is able to measure the increasing quality of the exam even though the measurement already started at a very high level considering the starting point of the FLE in 2011 (when one would expect the steepest learning curve), adding to the instrument’s validity argument.

The findings are incidentally not a measure of the overall quality of the FLE but a mosaic, as other potential sources of variance like the examiners, checklists and further contextual factors were not scrutinized and remained beyond the scope of this study. A potential source of bias in the study at hand lies in how local SP educators rated the quality of their own SPs’ role-portrayal, who were trained by themselves or their colleagues. This however constitutes a systematic error that would apply to all sites. Inter-rater agreement using the FAIR OSCE has so far not been reported and was beyond the scope of this study but should be addressed in future studies. Parallel assessment with the authenticity items of the MaSP [21] could contribute more to the validity argument of the FAIR OSCE. While the study was conducted in only one country in the context of only one exam, the fact it was performed in the context of a highly-standardized high-stakes federal licensing exam performed at five sites in two languages gives it a certain merit.

## Conclusions

SPs’ role-portrayal in a series of highly standardized high-stakes exams was shown to be of highest quality and increasing consecutively over all exam cohorts analyzed. Well-trained SPs observed with a valid instrument thus contribute to a standardized, high-quality clinical skills exam.

## Authors’ ORCIDs

- Kai P. Schnabel: [0000-0002-6977-2717]
- Daniel Bauer: [0000-0002-3337-3327]
- Tanja Hitzblech: [0000-0003-0876-0373]
- Beate G. Brem: [0000-0002-0551-9587]

## Acknowledgements

The authors would like to extend their gratitude towards all SP educators applying the instrument and collating the data during the exams that served as basis for the analyses.

## Competing interests

The authors declare that they have no competing interests.

## Supplementary Material

Comprehensive description of the clinical skills part of the Swiss Federal Licensing Exam in Medicine

English version of the FAIR OSCE instrument in its current form

## Figures and Tables

**Table 1:**
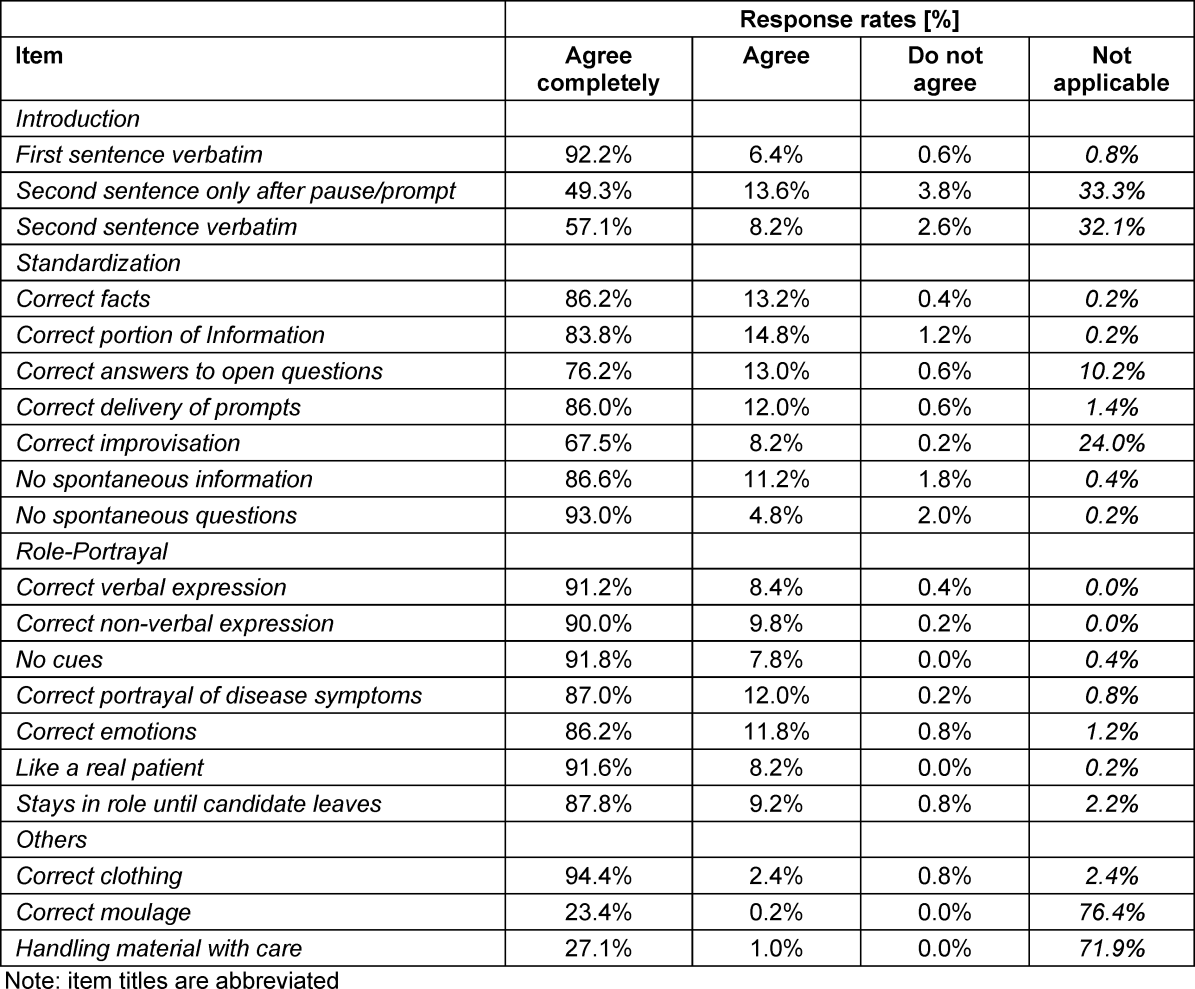
FAIR OSCE response rates as derived from the 2021 exam

**Table 2:**
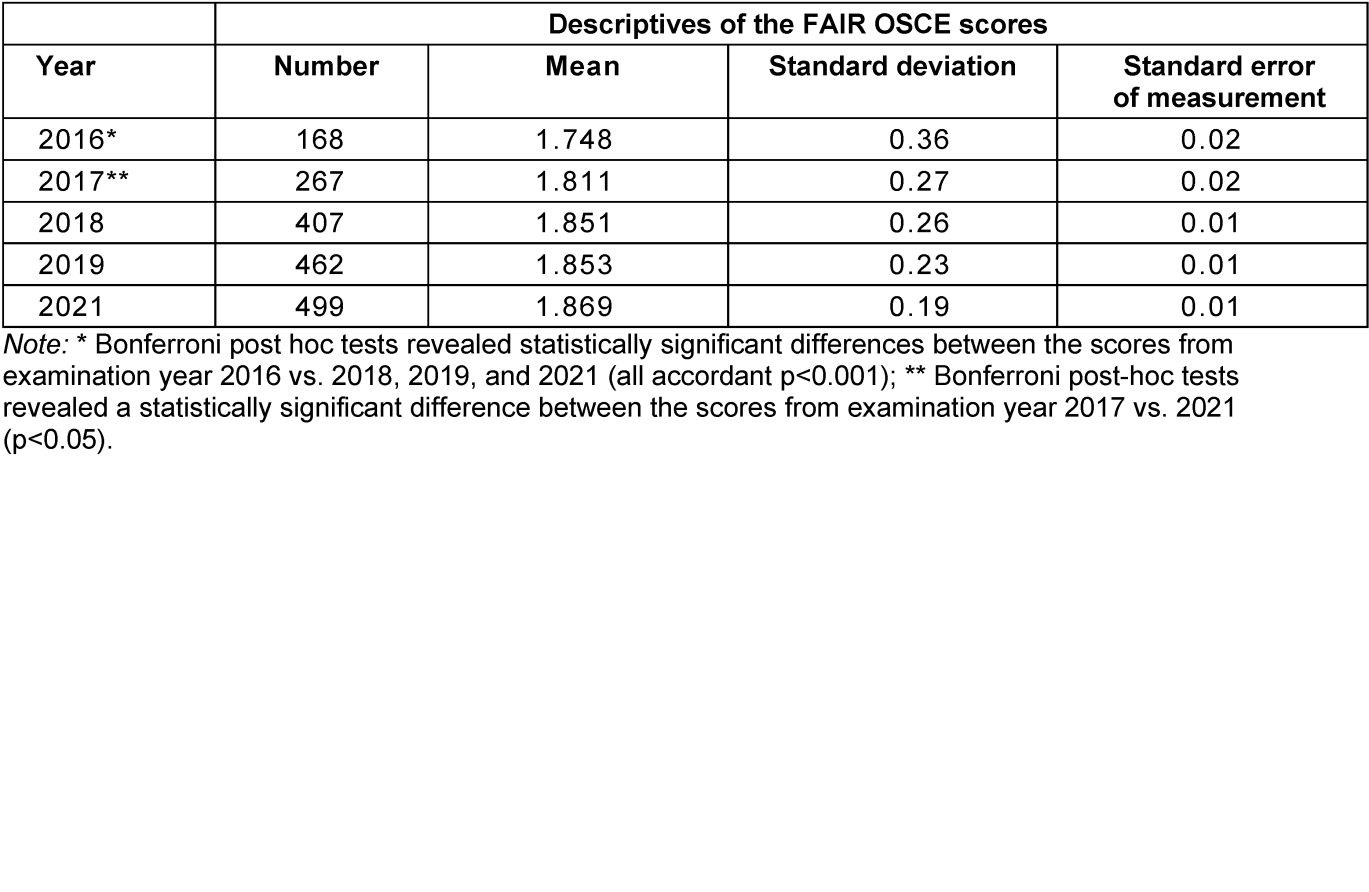
Statistics related to the FAIR OSCE scores per examination year
