# Supplementary material for: Simulated patients’ role-portrayal in the clinical skills part of the Swiss federal licensing exam is of high quality and improves further over time as measured with the FAIR OSCE instrument
Source: GMS J Med Educ. 2025 Feb 17;42(1):Doc12. doi: 10.3205/zma001736 (PMC12086251; doi:10.3205/zma001736)
Supplement: Comprehensive description of the clinical skills part of the Swiss Federal Licensing Exam in Medicine [file JME-42-12-s-001.pdf]

# Attachment 1: Comprehensive description of the clinical skills part of the Swiss Federal Licensing Exam in Medicine

## Comprehensive description of the Clinical Skills part of the Swiss Federal Licensing Exam in Medicine

reference period 2016-2021

### 1. Course background

| | |
|---|---|
| School/Institution | Medical faculties of the universities of Basel, Bern, Geneva, Lausanne and Zurich on behalf of the Swiss Federal Office of Public Health. |
| Course area | Medicine. |
| Curriculum years | Federal licensing exam after having graduated from a master program in medicine. |
| Medical education level | Graduation level. |
| Students' course year | Exam is typically taken in the 6th year (3 years Bachelor, 3 years Master). |
| Total number of students | 120-340 students per faculty. |
| Role of exam | High Stakes Federal Licensing Exam. |

### 2. OSCE design

| | |
|---|---|
| OSCE aims | To assess individual candidates' medical competence. |
| Role of exam | Summative. |
| Total number of students performing the OSCE | 800-1200 per year. |
| Maximum number of students per OSCE circuit | 15. |
| Briefing the students | Information supplied by the Federal Office of Public Health and onsite briefing through the faculties' person in charge. |
| Number of sites | 5 (Basel, Bern, Geneva, Lausanne, Zurich). |
| Number of exams | 5 (2016, 2017, 2018, 2019, 2021). |
| Number of parallel OSCEs (sequential/simultaneous) | 3 parallel (3 exam days). |
| Number of circuits | 2 per day (morning and afternoon). |
| Pre-test of stations (Pilot OSCE) | 0. |
| Subject and specialty areas being assessed | Competency-based assessment without attribution to specialties. The whole 6 years' content, as described in the national outcome framework (http://profilesmed.ch/), following a distinct blueprint. |
| Recording and scoring students' performance | Electronic checklist combining specific items and global ratings. |
| Criteria for pass/fail decision | Criterion-referenced post-hoc standard setting following the borderline regression method; the final pass/fail decisions rests with a federal examination board. |
| Number of Stations | 12. |
| Station details | 12 clinical stations, 2-3 additional rest stations. |
| Learning outcomes being assessed (Type of stations) | Clinical stations usually integrating history-taking, physical examination, management and communication competencies, according to the blueprint developed under consideration of PROFILES. |
| Station duration | 13 minutes, additional 2 minutes time to proceed to the following station. |
| Identical station duration | All stations have the same duration. |
| OSCE duration | 3 hours plus time from the 2-3 rest stations. |
| Number and details of teachers | Senior clinicians, SP experts and OSCE designers from the local sites. |
| Number and details of observers | SP experts, OSCE designers and administrative personnel, proctors from the local sites. |
| Number and details of examiners | Senior clinicians from the local sites, 1 per station. |
| Number and details of simulated patients | SP cast from and by the local SP programs, 1 per station. Usually a mix of professional and lay actors/ actresses. |
| Training Process for SP | Standardization of SP with local sites' SP experts in 2 sessions of 2 hrs each, with station-specific exemplary pre-taped video, all pre-exam. Additional 30 minutes in the morning of the exam day. |
| Training Process for examiners | Standardization for first-time examiners with briefing and rating of an exemplary pre-taped exam situation, pre-exam. Additional 30 minutes in the morning of the exam day. |
| Number of real patients | 0. |
| Number of stations using video, computer, mannequins or other | 0. |
| Feedback given to students, SPs. | No feedback to students beyond pass/fail. Feedback to SP as deemed appropriate by the local SP experts, e.g. based on FAIR OSCE observations. |
| Validity data | Rigorous quality control before, during and after the exam is in place. Additional information beyond what is implied from information supplied above cannot be disclosed. |

Above checklist based on *Madalena Patrício, Miguel Julião, Filipa Fareleira, Meredith Young, Geoffrey Norman & António Vaz Carneiro (2009) A comprehensive checklist for reporting the use of OSCEs, Medical Teacher, 31:2, 112-124, DOI: 10.1080/01421590802578277*

Attachment 1 to Schnabel KP, Bauer D, Schmitz FM, Hitzblech T, Brem BG. *Simulated patients' role-portrayal in the clinical skills part of the Swiss federal licensing exam is of high quality and improves further over time as measured with the FAIR-OSCE instrument.* GMS J Med Educ. 2025;42(2):Doc12. DOI: 10.3205/zma001736
