# Supplementary material for: Simulated patients’ role-portrayal in the clinical skills part of the Swiss federal licensing exam is of high quality and improves further over time as measured with the FAIR OSCE instrument
Source: GMS J Med Educ. 2025 Feb 17;42(1):Doc12. doi: 10.3205/zma001736 (PMC12086251; doi:10.3205/zma001736)
Supplement: English version of the FAIR OSCE instrument in its current form [file JME-42-12-s-002.pdf]

Attachment 2: English version of the FAIR OSCE instrument in its current form

UNIVERSITÄT BERN

FAIR OSCE

Focused Assessment of Interactive Role-play in Objective Structured Clinical Examinations

Faculty: ____________________ Rater: ____________________

Exam: ____________________ Date: ____________________

Standardized Patient: ____________________ Time: ____________________

Role: ____________________

Introduction

The SP ….	Agree Completely	Agree	Do no Agree	Not applicable
… delivers the first sentence verbatim.	☐	☐	☐	☐
… delivers the second sentence after a pause or answering an open question.	☐	☐	☐	☐
… delivers the second sentence verbatim.	☐	☐	☐	☐

Comments

Deliver of Information

The SP ….	Agree Completely	Agree	Do no Agree	Not applicable
… delivers correct facts.	☐	☐	☐	☐
… delivers appropriate amounts of information.	☐	☐	☐	☐
… answers correctly to open questions.	☐	☐	☐	☐
… delivers scripted prompts (questions, information, etc. Timely and correct.)	☐	☐	☐	☐
… improvises appropriately, if necessary	☐	☐	☐	☐
… does not give relevant verbal information spontaneously, Unless scripted.	☐	☐	☐	☐
… does not ask spontaneous questions, unless scripted.	☐	☐	☐	☐

Comments

Attachment 2 to Schnabel KP, Bauer D, Schmitz FM, Hitzblech T, Brem BG. *Simulated patients' role-portrayal in the clinical skills part of the Swiss federal licensing exam is of high quality and improves further over time as measured with the FAIR-OSCE instrument.* GMS J Med Educ. 2025;42(2):Doc12. DOI: 10.3205/zma001736

b
UNIVERSITÄT
BERN

Portrayal

The SP ….	Agree Completely	Agree	Do no Agree	Not applicable
… shows appropriate verbal expression.	☐	☐	☐	☐
… shows appropriate non-verbal expression.	☐	☐	☐	☐
… does not give non-verbal clues unless scripted.	☐	☐	☐	☐
… presents medical condition.	☐	☐	☐	☐
… presents emotions correct.	☐	☐	☐	☐
… plays the role in a way that he/she appears to be a real patient.	☐	☐	☐	☐
… stays in a role until candidate leaves the room.	☐	☐	☐	☐

Comments

Others

The SP ….	Agree Completely	Agree	Do no Agree	Not applicable
… wears appropriate clothes and accessories.	☐	☐	☐	☐
… wears appropriate moulage.	☐	☐	☐	☐
… handles materials and models carefully and correctly.	☐	☐	☐	☐

Comments

Attachment 2 to Schnabel KP, Bauer D, Schmitz FM, Hitzblech T, Brem BG. *Simulated patients' role-portrayal in the clinical skills part of the Swiss federal licensing exam is of high quality and improves further over time as measured with the FAIR-OSCE instrument.* GMS J Med Educ. 2025;42(2):Doc12. DOI: 10.3205/zma001736
